# Nail Involvement in Patients with Psoriatic Arthritis in Northern Iran

**DOI:** 10.1155/2018/4608490

**Published:** 2018-10-15

**Authors:** O. Zargari, E. Kazemnezhad Leyli, S. Z. Azimi

**Affiliations:** ^1^Department of Dermatology, Skin Research Center, Shahid Beheshti University of Medical Sciences, Tehran, Iran; ^2^Department of Biostatistics, Guilan University of Medical Sciences, Rasht, Iran; ^3^Department of Dermatology, Skin Research Center, Guilan University of Medical Sciences, Rasht, Iran

## Abstract

**Background:**

Psoriatic arthritis (PsA) results in an increased burden of psoriasis and impairs both quality of life and an individual's functional capacity. The relationship between nail involvement and PsA in psoriasis is not fully characterized.

**Aim:**

To evaluate the frequency and characteristics of nail involvement in psoriatic patients and to assess the relationship with joint involvement.

**Methods:**

A total of 197 patients with moderate-to-severe psoriasis were consecutively invited to participate in this cross-sectional study. The patients are divided into two groups: those with and those without psoriatic arthritis.

**Results:**

69.5% of psoriatic (137 out of 197) patients had nail involvement. The most common nail abnormality was onycholysis, followed by pitting and oil droplet changes. Nail involvement was more common in patients with psoriatic arthritis (82.1% versus 57.8%, p=0.001).

**Conclusion:**

Nail involvement is commonly associated with PsA. Onycholysis, splinter hemorrhage, and oil drop were significantly more common in the PsA group as opposed to patients with just skin findings. In general, psoriatic patients with arthritis had more severe disease.

## 1. Introduction

Psoriasis is a chronic inflammatory disease affecting up to 3% of the general population. It has a substantial negative impact on health-related quality of life (HRQOL), especially when it involves visible areas of the body such as the face, hands, and nails [1]. Psoriatic arthritis (PsA) has been defined as inflammatory arthritis, usually seronegative, associated with psoriasis [1]. The exact prevalence of PsA is unknown but up to 30% of patients with psoriasis develop psoriatic arthritis [2, 3].

A substantial number of psoriatic patients have nail involvement, varying from 10% to 80%, with an estimated lifetime incidence of 80–90% [4–6].

Nail involvement may be considered an indicator for patients at risk for future psoriatic joint damage [7, 8]; however, this relationship has not been proven [9]. The aim of this study was to assess the overall prevalence of nail involvement in patients with moderate-to-severe psoriasis and to achieve more details on the possible relationship between nail psoriasis and PsA.

## 2. Materials and Methods

### 2.1. Patients

This cross-sectional study was conducted from January 2017 to December 2017. During this period, all eligible patients with moderate-to-severe psoriasis who attended to our psoriasis clinic and were candidates for systemic therapy were enrolled in the study. Moderate-to-severe psoriasis was defined as body surface area (BSA) >10% or psoriasis area and severity index (PASI) >10 [10]. The study was approved by the Shahid Beheshti institutional board committee. Informed consent was obtained from each subject.

A detailed history was recorded for all subjects, including gender, age, age at the time of onset of the disease, phenotype of psoriasis, disease duration, extent, and severity of the disease. We diagnosed psoriasis according to the clinical findings, and if necessary, a skin biopsy was performed. All patients underwent dermatological examination by a dermatologist (O.Z.). The following nail changes were recorded: pitting, crumbling, onycholysis, oil drop, subungual hyperkeratosis, and splinter hemorrhage.

Psoriasis skin severity was assessed by using psoriasis area and severity index (PASI) at the time of the physical examination. Mycological investigations were performed in patients with nail changes suspicious for fungal infection such as onychorrhexis, hyperkeratosis, thickening, or crumbling. Nail clippings and subungual scrapings were collected. One part of nail specimens was exposed to direct microscopic examination with 10% potassium hydroxide aqueous solution and the other part was cultured on Sabouraud's dextrose agar medium with cycloheximide. Also, other causes of nail changes, like congenital and traumatic dystrophy were excluded from the study. Isolated nail psoriasis patients were not included in the study.

On the basis of musculoskeletal findings, patients with arthralgia or other joint symptoms were referred to a rheumatologist. The diagnosis of psoriatic arthritis was established in accordance with the Classification Criteria for Psoriatic Arthritis (CASPAR).

### 2.2. Statistical Analysis

Statistical analysis was performed using software SPSS 18.0 (SPSS Inc., IBM Corporation, Armonk, New York). Categorical variables were expressed as frequencies and percentages and analyzed by Chi-square test or Fisher's exact test. Normality of variables was verified by Kolmogorov-Smirnov test. Continuous variables were given as means (SD) or medians (range). Student t-test was used for continuous variables with normal distribution. For continuous variables with an abnormal distribution, the Mann–Whitney test was performed. Statistical significance was considered at a level of 5% (p<0.05) for all tests.

## 3. Results

In total, 197 patients participated in the study. There was a slight female preponderance in our sample with 54 % (n = 107) female patients and 46% (n = 90) male patients.

The mean duration of the disease was 15 years (SD: 11 years; ranging from one month to 46 years) and the mean age of disease onset was 30 ±17 years. The subjects were divided into two groups: those with and those without psoriatic arthritis. In the study population, 95 patients (48%) had psoriatic arthritis. Of these, 56 patients were women and 39 patients were men (Table 1).

We found no significant difference between the two groups in terms of age, gender, the presence of comorbidities, alcohol intake and smoking. However, patients with PsA had lower median age of onset [26.91 ± 16.65 versus 31.87 ± 17.06 years, p=0.04] and longer disease duration [17.24 ± 10.79 versus 13.06 ± 10.42, p=0.006]. Also, the frequency of facial involvement was higher in those with PsA (74.7% versus 54.9%, p=0.001). In general, patients with PsA had more severe psoriasis reflected by higher median PASI scores [20.05 ± 11.54 versus 15.37 ± 8.97, p=0.016]. Also, positive family history for psoriasis was more often reported in those with PsA (49.5% versus 34.3%, p=0.03).

Overall, methotrexate was the most used medication among patients (88.3%). Patients in PsA group had a higher rate for the history of using different systemic treatments.

Prevalence of plaque-type psoriasis was significantly higher in patients with PsA (Table 2).

**Figure 1:**
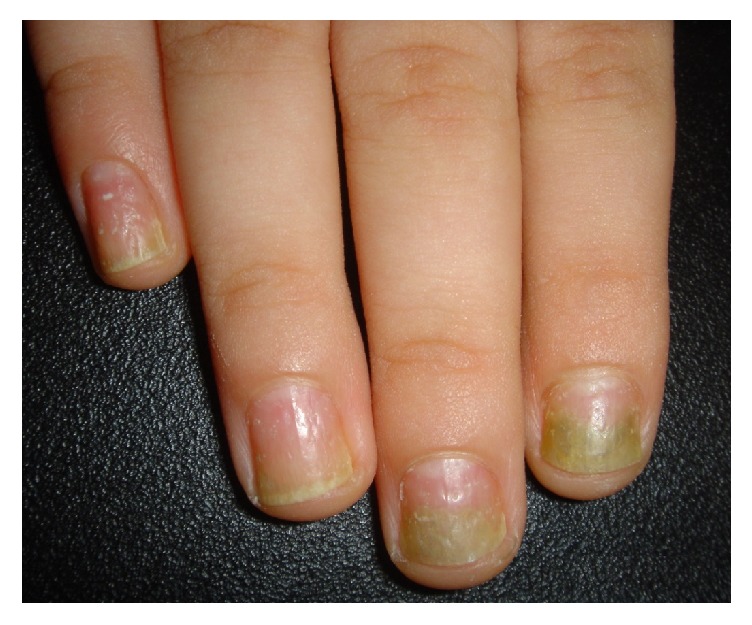
Pitting and onycholysis in a psoriatic patient.

Nail involvement was present in 69.5% (137 out of 197) of patients. The most common nail abnormality was onycholysis, followed by pitting and oil drop (68.4%, 34.7%, and 30.5%, respectively). Nail changes were more common in patients with PsA (82.1% vs. 57.8%, p=0.001). Prevalence of onycholysis, splinter hemorrhage, and oil drop were significantly higher in PsA patients in comparison with patients without psoriatic arthritis (Table 3).  Figure 1, illustrates pitting and onycholysis in a patient.

## 4. Discussion

The prevalence of nail changes in psoriatic patients reportedly presents great variety in the literature. Nail involvement in psoriasis usually is divided into two major groups: (a) signs of involvement of nail matrix including pitting, leukonychia, red spots of the lunula, transverse grooves (Beau's lines), and crumbling of the nail plate and (b) signs of involvement of the nail bed which present as oil-drop discoloration, splinter hemorrhages, subungual hyperkeratosis, and onycholysis [5].

The most common psoriasic nail features were dissimilar in different studies. In a report by Kyriakou et al., oil droplet changes were the most common finding [11]. In another study, by Kaur et al. nail pitting was the most common finding [12]. On the other hand, there are reports showing that subungual hyperkeratosis is the most common feature [13]. Our study was in concordance with Grover's paper [14] that the most common nail finding was onycholysis, which was present in 54.8% of our patients (108 out or 197) and significantly was more prevalent in the PsA group.

Pits can be seen in normal individuals as well as those with chronic eczema, alopecia areata, and lichen planus. Therefore, seeking other psoriatic nail features would be helpful for the identification of psoriatic nail pitting. Also, it is suggested that pits in patients with nail psoriasis are typically deeper than those observed in association with other dermatological conditions [10, 15].

Forty-eight percent of our patients had PsA. This figure is slightly higher than previous studies, which suggest a prevalence of around 30% [16, 17]. The higher rate of PsA in our patients is probably due to the selection of relatively more severe cases in this study.

Our study revealed that the prevalence of nail changes is higher in PsA, than has been suggested by some previous observations [18, 19]. We found that onycholysis, oil drop, and splinter hemorrhage, in particular, were significantly more prevalent in PsA patients. Fonseca et al. found no statistical difference in NAPSI (Nail Psoriasis Severity Index) values among patients with or without psoriatic arthritis [20]. Perhaps, the type of nail involvement is more important than the severity of nail changes in terms of correlation with PsA.

The pathophysiology of nail dystrophy has been postulated to be more closely associated with joint symptoms than with skin symptoms [21]. However, there is still no clear explanation for this association and some believe that this association is limited only to distal interphalangeal (DIP) joints [22]. The suggested theories are (a) having a common (and yet unknown) autoantigen in both nail apparatus and synovial membrane, (b) anatomic connection between nail structures and DIP joints, and (c) the common role of trauma (Koebner phenomenon) in both nail and arthropathic psoriasis [20, 23, 24].

In this study, patients with PsA had more severe psoriasis and an earlier age of onset and longer duration than those without PsA. Also, they had a higher rate of facial involvement. To our knowledge, this association has not been reported previously and further studies are needed to corroborate this association.

We excluded all psoriatic patients with onychomycosis from the study after positive direct examination, KOH smear and/or fungal culture. However, onychomycosis may present with clinical features similar to nail psoriasis. In addition, it is anticipated that the prevalence of onychomycosis is about 4.6 to 30% of psoriatic patients with nail involvement [25, 26].

Our study had certain limitations. One of them was lack of information about the subtypes of PsA. Also, we have not recorded the severity of nail involvements. Furthermore, previous medications may have interfered with the degree of nail changes in our patients.

## 5. Conclusion

In summary, we have shown that there is an association between nail involvement and PsA and this association is most significant for onycholysis. Also, patients with PsA have a higher probability of positive family history for psoriasis, earlier onset, longer duration, more severe psoriasis and a higher rate of facial involvement. It should be kept in mind that while there is a strong association between PsA and nail changes, every psoriasis patient should be asked about joint symptoms even if they have no nail changes.

## Figures and Tables

**Table 1:** Demographic and clinical characteristics of patients.

	**All patients** **(N= 197)**	**Patients without PsA** **(N=102)**	**Patients with PsA** **(N=95)**	**P Value**
**Age **(Year, mean ±SD)	44.56 ± 14.73	44.93 ± 16.06	44.16 ± 13.24	0.71
**Sex**				
Male	90 (45.7%)	51 (50%)	39 (41.1%)	0.21
Female	107 (54.3%)	51 (50%)	56 (58.9%)	
**Age at onset **(Year, mean ±SD)	29.48 ± 17.00	31.87 ± 17.06	26.91 ± 16.65	**0.04**
**Duration of psoriasis **(Year, mean ±SD)	15.08 ± 10.78	13.06 ± 10.42	17.24 ± 10.79	**0.006**
**Positive family history**	82 (41.6%)	35 (34.3%)	47 (49.5%)	**0.03**
**BMI, **(kg/m^2^; mean ± SD)	28.97 ± 6.33	28.11± 6.05	29.95 ± 6.52	**0.05**
**PASI**	17.67 ± 10.53	15.37 ± 8.97	20.05 ± 11.54	**0.01**
**Nail involvement**	137 (69.5%)	59 (57.8%)	78 (82.1%)	**0.001**
**Face involvement**	127 (64.5%)	56 (54.9%)	71 (74.7%)	**0.004**
**Genital involvement**	126 (64%)	59 (57.8%)	67 (70.5%)	0.06
**Smoking**	32 (16.2%)	19 (18.6%)	13 (13.7%)	0.35
**Alcohol**	26 (13.2%)	14 (13.7%)	12 (12.6%)	0.82
**Comorbidities**				
**DM**	36 (18.3%)	17 (16.7%)	19 (20%)	0.54
**HTN**	36 (18.3%)	17 (16.7%)	19 (20%)	0.54
**CVD**	11 (5.6%)	6 (5.9%)	5 (5.3%)	0.85
**HLP**	37 (18.8%)	20 (19.6%)	17 (17.9%)	0.76
**Prior Treatments**				
**Methotrexate**	174 (88.3%)	85 (83.3%)	89 (93.7%)	**0.024**
**Acitretin**	122 (61.9%)	62 (60.8%)	60 (63.2%)	0.73
**CyA**	73 (37.1%)	27 (26.5%)	46 (48.4%)	**0.001**
**Phototherapy**	36 (18.3%)	13 (12.7%)	23 (24.2%)	**0.04**
**Systemic steroids**	21 (10.7%)	7 (6.9%)	14 (14.7%)	0.07
**Biologics**	64 (32.5%)	18 (17.6%)	46 (48.4%)	**0.001**

BMI: body mass index, PASI: psoriasis and area severity index, DM: diabetes mellitus, HTN: hypertension, CVD: cardio vascular disease, HLP: hyperlipidemia, and CyA: cyclosporine A.

**Table 2:** Psoriasis phenotypes in the groups studied.

**Type of Psoriasis**	**All patients** **(N= 197)**	**Patients without PsA** **(N=102)**	**Patients with PsA** **(N=95)**	**P Value**
**Chronic plaque **	181 (91.9%)	90 (88.2%)	91 (95.8%)	**0.05**
**Pustular**	2 (1%)	1 (1%)	1 (1.1%)	0.96
**Palmoplantar**	33 (16.8%)	19 (18.6%)	14 (14.7%)	0.53
**Flexural **	13 (6.6%)	8 (7.8%)	5 (5.8%)	0.46
**Guttate**	32 (16.2%)	13 (12.7%)	19 (20%)	0.16
**Erythrodermic**	5 (2.5%)	2 (2%)	3 (3.2%)	0.59

**Table 3:** Morphologic features of nail changes.

**Nail Features **	**All patients** **(N= 197)**	**Patients without PsA** **(N=102)**	**Patients with PsA** **(N=95)**	**P Value**
**Pitting**				0.34
**Yes**	62 (31.5%)	29 (28.4%)	33 (34.7%)	
**No**	135 (68.5%)	73 (71.6%)	62 (65.3%)	
**Total Dystrophy**				0.71
**Yes**	3 (1.5%)	0 (0)	3 (3.2%)	
**No**	194 (98.5%)	102 (100%)	92 (96.8%)	
**Onycholysis**				**0.001**
**Yes**	108 (54.8%)	43 (42.2%)	65 (68.4%)	
**No**	89 (45.2%)	59 (57.8%)	30 (31.6%)	
**Oil Drop**				**0.013**
**Yes**	45 (22.8%)	16 (15.7%)	29 (30.5%)	
**No**	152 (77.2%)	86 (84.3%)	66 (69.5%)	
**Subungual hyperkeratosis**				0.69
**Yes**	19 (9.6%)	9 (8.8%)	10 (10.5%)	
**No**	178 (90.4%)	93 (91.2%)	85 (89.5%)	
**Splinter hemorrhage**				**0.05**
**Yes**	12 (6.1%)	3 (2.9%)	9 (9.5%)	
**No**	185 (93.9%)	99 (97.1%)	86 (90.5%)	
**Longitudinal ridging**				0.28
**Yes**	4 (2%)	1 (1%)	3 (3.2%)	
**No**	193 (98%)	101 (99%)	92 (96.8%)	

## Data Availability

The data and analysis used to support the findings of this study are available from the corresponding author upon request.
